# Acute respiratory distress syndrome and acute myocarditis developed in a previously healthy adult with influenza B

**DOI:** 10.1186/s12890-015-0163-3

**Published:** 2016-01-04

**Authors:** Hsu-Liang Chang, Jui-Feng Hsu, Ying-Ming Tsai, Shang-Yi Lin, Hsuan-Fu Kuo, Chih-Jen Yang

**Affiliations:** Department of Internal Medicine, Kaohsiung Municipal Ta-Tung Hospital, Kaohsiung Medical University, Kaohsiung, Taiwan; Division of Pulmonary and Critical Medicine, Department of Internal Medicine, Kaohsiung Medical University Hospital, Kaohsiung Medical University, #100, Tzyou 1st Road, Kaohsiung 807, Taiwan; School of Medicine, College of Medicine, Kaohsiung Medical University, Kaohsiung, Taiwan

**Keywords:** Influenza B, Myocarditis, Acute respiratory distress syndrome

## Abstract

**Background:**

Influenza B virus infection is generally considered to be mild and is rarely associated pulmonary cardiovascular involvement in adults. However fatal complications may occur.

**Case presentation:**

A 43-year-old previously healthy Taiwanese male came to our emergency department due to high fever, chills, general malaise and myalgia for about 4 days. An influenza rapid test from a throat swab was negative. Chest radiography showed mild left lung infiltration and levofloxacin was prescribed. However, progressive shortness of breath and respiratory failure developed 48 h later after hospitalization. Emergent intubation was performed and he was transferred to the intensive care unit where oseltamivir (Tamiflu, Roche) 75 mg orally twice daily was given immediately. In the intensive care unit, cardiac catheterization revealed normal coronary arteries. However, a markedly elevated cardiac enzyme level (Troponin I level was up to 71.01 ng/ml), a positive cardiac magnetic resonance imaging findings and no coronary artery stenosis led to the diagnosis of acute myocarditis. Subsequent real-time polymerase chain reaction of endotracheal aspirates was positive for influenza B. His condition gradually improved and he was successfully weaned from the ventilator on day 22. He was discharged without prominent complications on day 35.

**Conclusion:**

Influenza B infection is not always a mild disease. Early detection, early administration of antiviral agents, appropriate antibiotics and best supportive care, is still the gold standard for patients such as the one reported.

## Background

Influenza is a common upper respiratory infection associated with a high spreading rate and low mortality. Clinically, influenza is usually a self-limiting disease but may cause uncommon but fatal complications, including rapidly progressive pneumonia, development of acute respiratory distress syndrome (ARDS) [1, 2] and sometimes, lethal cardiac complications such as acute myocarditis and even cardiogenic shock [3–6]. Because influenza A is so important and has caused many endemic outbreaks, the significance of influenza B may have been underestimated [1]. Herein, we present a very rare case who is a previously healthy adult patient with influenza B and rapidly experienced both ARDS and acute myocarditis.

## Case Presentation

A previously healthy 43-year-old man was sent to our emergency department, with a high fever, chills, throat pain, myalgia, and poor appetite for about four days.

**Fig. 1:**
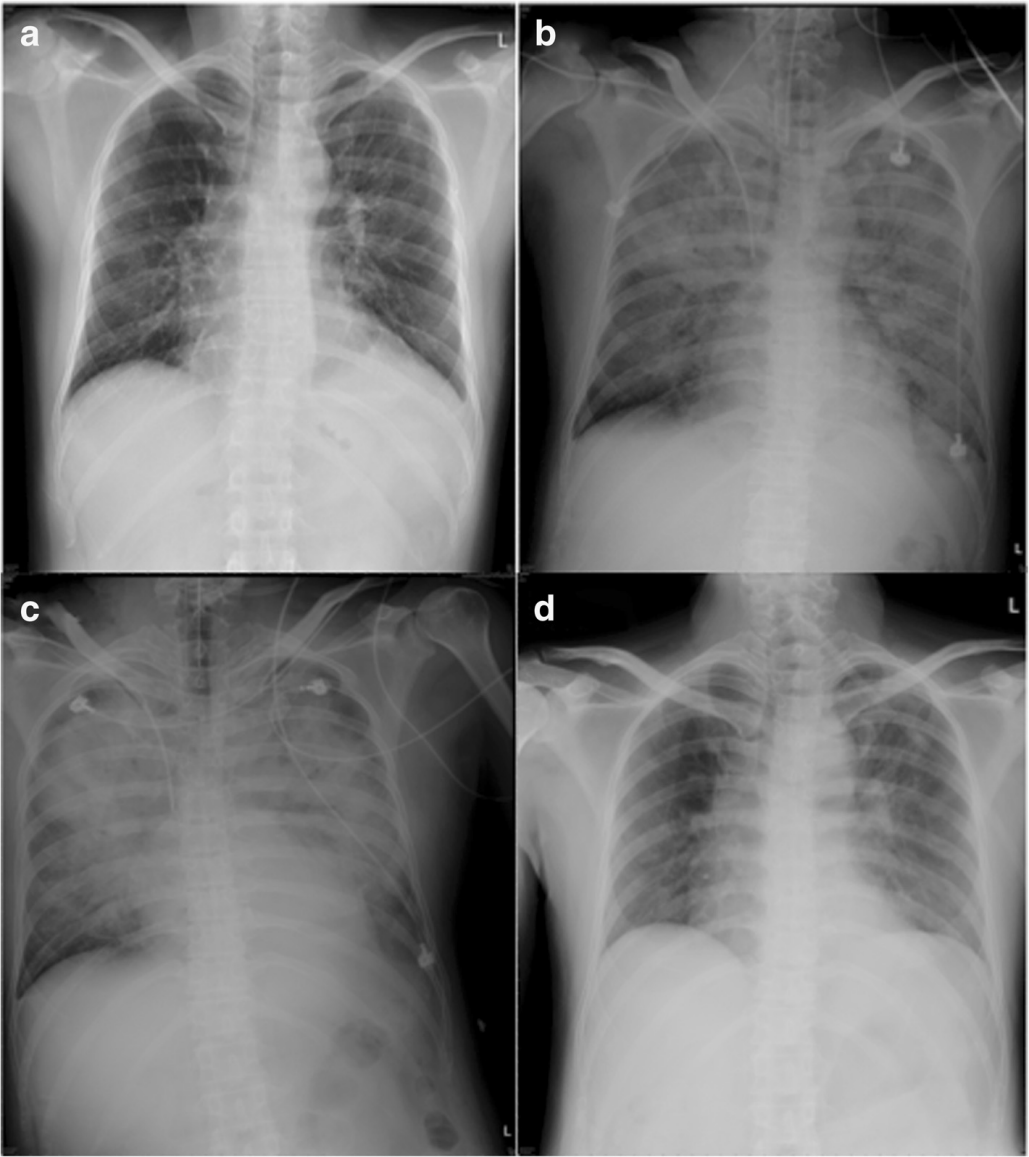
Initial chest radiography demonstrated mild pulmonary infiltration over the left lung on day 1 (**a**). A portable chest X-ray revealed prominent bilateral infiltrates at 48 h later after admission to our hospital (**b**), progressed to worse picture on day 4 (**c**) and recovery to near-normal x ray on day 22 (**d**)

His initial blood temperature, pulse rate, and blood pressure were 40 °C, 112 beats/min, and 120/71 mmHg, respectively. Chest radiography revealed mild infiltrates in the left lung field (Fig. 1a). No leukocytosis (white blood count 7380/ul; normal range: 4140 ~ 10520/ul) but predominant neutrophils (neutrophil: 85.8 %, normal range: 41.8 ~ 70.8 %), thrombocytopenia (platelets 92000/ul, normal range 160000 ~ 370000/ul), and high C-reactive protein 77.30 mg/L(normal range <5 mg/L), aspartate aminotransferase 384 u/L(normal range:10 ~ 42 u/L), alanine aminotransferase 291 u/L(normal range: 10 ~ 40 u/L), and troponin I 0.06 ng/ml levels (normal range < 0.04 ng/ml). A rapid influenza test using a specimen from a nasopharyngeal swab was negative. In addition, urine Streptococcus Ag, Legionellae serotype I and serum Mycoplasma IgM were all negative.

Rapidly, his general condition worsened with chest tightness, cold sweating, and dyspnea. Electrocardiography (ECG) showed sinus tachycardia and the followed cardiac enzyme tests revealed a creatine kinase (CK) level of 1593 IU/L(normal range 26 ~ 174 IU/L), a CK-MB level of 45.2 IU/L(normal range: 0.4 ~ 6.3 IU/L) and a troponin I level up to 71.01 ng/ml (normal range < 0.04 ng/ml). The B-type natriuretic peptide level was 104 pg/ml (BNP < 100 pg/ml). Subsequent chest radiography revealed diffuse bilateral opacities (Fig. 1b and c). He was then transferred to the intensive care unit (ICU) for emergency intubation and mechanical ventilator support (48 h later after admission) due to acute respiratory failure. He was treated with oseltamivir 75 mg orally twice daily for 5 days from day 4 even though the rapid influenza test was negative. Finally, reverse transcription polymerase chain reaction (RT-PCR) from endotracheal aspirates showed positive results for influenza B based on the report of Centers for Disease Control, Taiwan.

In the ICU, because of his high serum cardiac enzyme, myocardia infarction was suspected and coronary angiography was arranged soon but it revealed no prominent stenosis. Cardiac echography showed normal chamber size, normal LV segmental wall motion without significant valvular defect and the ejection fraction of left ventricle was about 60 %. The result is not compatible with heart failure.

**Fig. 2:**
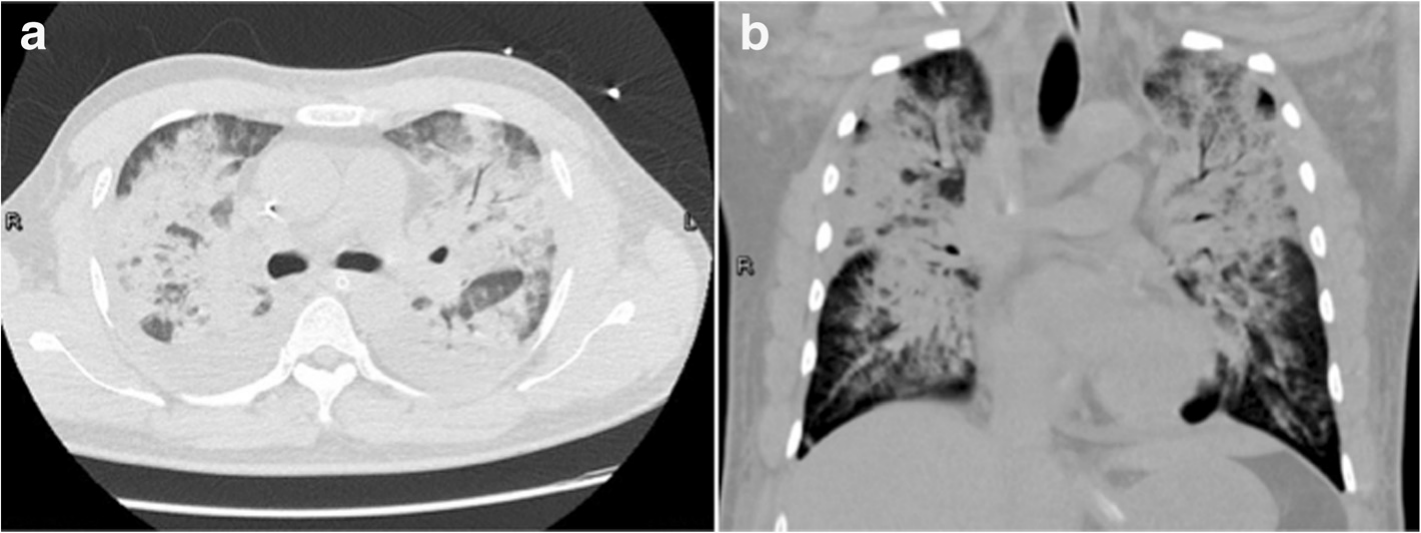
Chest CT showed diffuse air space lesions with air-bronchograms in both lungs (**a**) and (**b**)

**Fig. 3:**
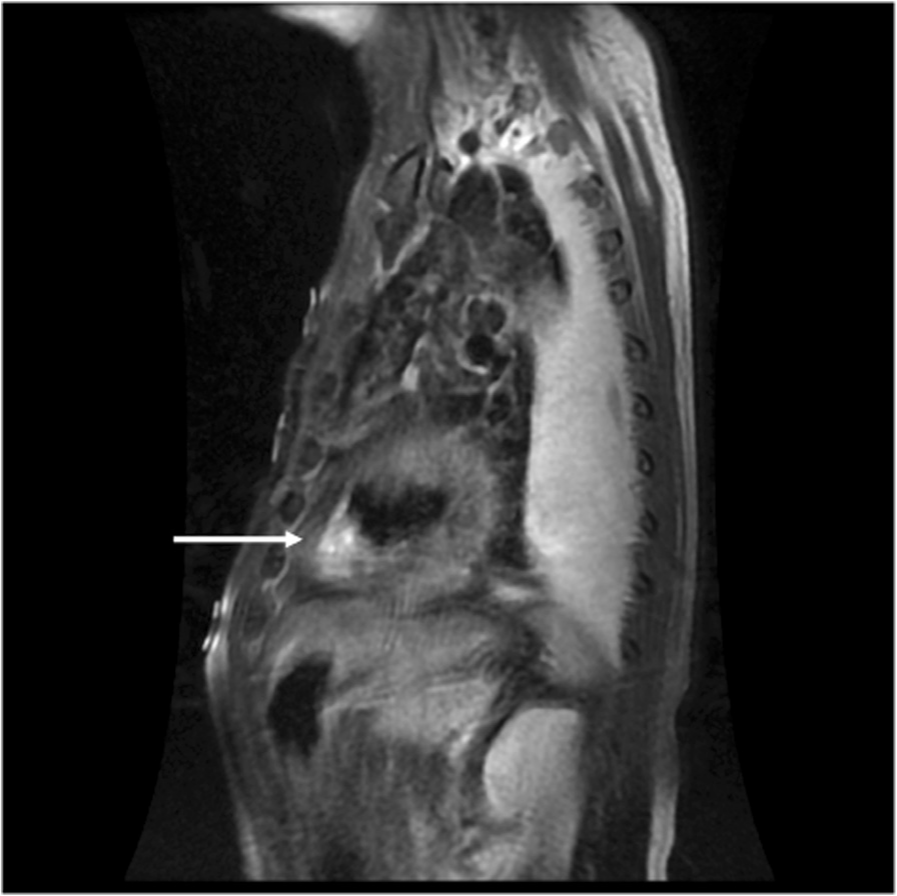
T2-weighted contrast-enhanced MRI view revealed gadolinium enhancement in the left ventricular wall, especially at the anterior-inferior wall of the left ventricle, consistent with myocarditis (a)

**Fig. 4:**
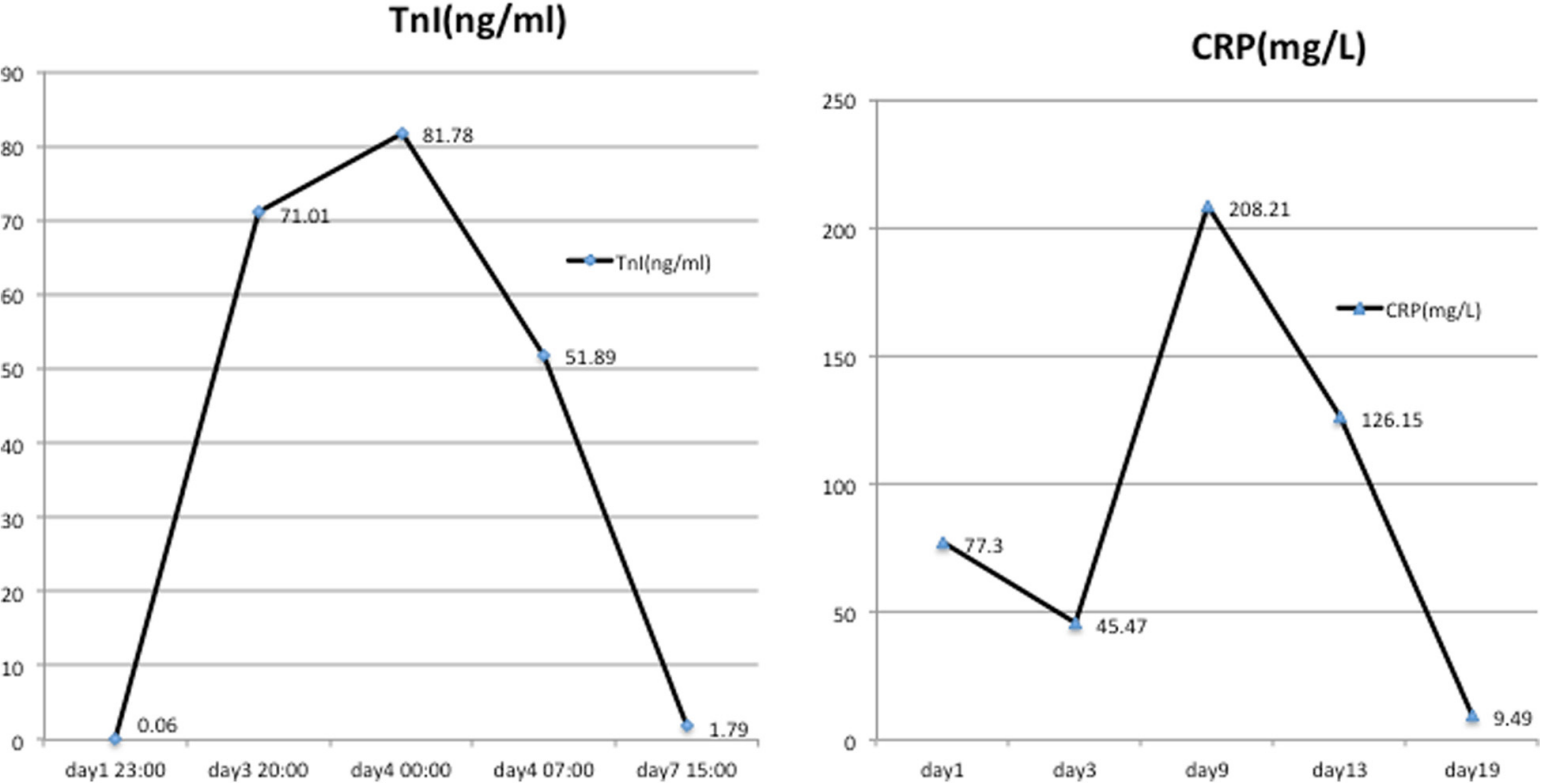
The time course of troponin I and CRP during the hospitalization

Besides, the chest x ray and chest CT both showed diffuse lung opacities. Taken together the above clinical evidence, influenza B related acute respiratory distress syndrome was diagnosed (Fig. 2a, b). Initially, empiric antibiotic with levofloxacin was administered but imipenem/cilastatin 500 mg q6h was then administered because of his rapidly worsening ARDS after being transferred to the ICU. His conditions gradually improved and the endotracheal tube was successfully removed on day 22 (Fig. 1d), after which he returned to an ordinary ward. Cardiac magnetic resonance imaging (MRI) showed abnormal enhancement of left ventricular wall, especially at anterior-inferior wall of the left ventricle (Fig. 3), consistent with acute myocarditis. Fortunately, he was discharged without prominent complications on day 35. The time course of troponin I and CRP were demonstrated in Fig. 4.

## Discussions

This is maybe the first report of influenza B with ARDS and myocarditis according the Pubmed search. We tried to show the progress note of the patient.

The influenza B virus usually causes mild disease, however it may induce rare but fatal complications such as myocarditits [2–4], myositis [5], ARDS [6]. The extension of influenza viral infection to the lower respiratory tract may result in viral pneumonia which can spread into all of the internal organs. The most rapid and reliable diagnosis of influenza was rapid test via nasopharyngeal swab. However, the presented patient showed negative to initial influenza rapid test and missed the early detection and early treatment of influenza B. In fact, the performance of the different rapid tests kits varied with sensitivities between 71.0 and 82.1 % and between 37.2 and 47.7 % for detecting influenza A and B, respectively. The specificity of all rapid tests was 100 % [7].

An autopsy report of patients who died from influenza B infection indicated that death often followed a remarkably rapid clinical progression, regardless of demonstrable bacterial pneumonia, and about 50 % of all patients died within 3 days after the onset of illness, with more than 70 % dying within 4 days [8]. Both primary viral pneumonia and secondary bacterial pneumonia can cause acute lung injury and even ARDS, characterized by the influx of protein-rich fluid into the air spaces as a consequence of increased permeability of the alveolar-capillary barrier. ARDS is different from congestive heart failure but always confused. According to the Berlin ARDS definition, ARDS was defined as occurred within 1 week, a chest radiograph or computed tomography scan showing bilateral opacities not fully explained by effusions, lobar/lung collapse, or nodules and respiratory failure not fully explained by cardiac failure or fluid overload [9]. Based on the report of cardiac echography, no evidence of heart failure at that moment though myocarditis occurred. In addition, PaO2/FiO2 was below 200 mmHg. Therefore, we treated the patient with ARDS strategies, such as lung protective strategy and intensive care.

An autopsy report of patients who died from influenza B virus infection revealed that up to 69 % of the patients had myocardial injury, predominantly in patients aged less than 18 years. Cases of adults with influenza B and myocarditis have rarely been reported. Clinical manifestations of myocarditis range from asymptomatic ECG abnormalities, arrhythmia to cardiogenic shock. Some patients have rapidly progressive cardiomyopathy, ventricular tachycardia and even cardiac death [10]. The most common ECG abnormality being sinus tachycardia with non-specific ST-T wave changes, which was seen in the present case. Troponin I has been reported to have a high specificity (89 %) but limited sensitivity (34 %) for the detection of myocyte injury in the diagnosis of myocarditis [11]. An endomyocardial biopsy is the gold standard for the diagnosis of myocarditis but rarely performed. Recently, published guidelines have suggested that cardiac MRI should be performed in patients with suspected myocarditis [12]. MRI has the unique potential to visualize tissue changes and can detect the characteristic changes in myocarditis including intracellular and interstitial edema, capillary leakage, hyperemia and, in more severe cases, cellular necrosis and subsequent fibrosis [13]. A combination of T1- and T2-weighted images with early and late gadolinium enhancement have been reported to provide the best combination of sensitivity and specificity. Our presented patients had extremely high cardiac enzyme and positive for myocardial injury based on cardiac MRI and his heart function is relatively normal.

Fortunately, the patient was discharged without any prominent complications after aggressive management with antiviral agents, appropriate antibiotics and ventilator support.

### Informed consent

Written informed consent was obtained from the patient for publication of this case report and any accompanying images. A copy of the written consent is available for review by the Editor of this journal.
